# IDIOPATHIC CALCINOSIS CUTIS

**DOI:** 10.4103/0019-5154.57624

**Published:** 2009

**Authors:** Padmavathy Lanka, Lakshmana Rao Lanka, N Ethirajan, B Krishnaswamy, U Manohar

**Affiliations:** *From the Urban Health Center, Division of Community Medicine, Rajah Muthiah Medical College, Annamalai University, Annamalai Nagar, Chidambaram - 608 002, Tamil Nadu, India.*; 1 *From the Division of Patholgy, Rajah Muthiah Medical College, Annamalai University, Annamalai Nagar, Chidambaram - 608 002, Tamil Nadu, India.*; 2 *From the Community Medicine, Rajah Muthiah Medical College, Annamalai University, Annamalai Nagar, Chidambaram - 608 002, Tamil Nadu, India.*

Sir,

A 10-year-old boy, born of a consanguineous marriage, presented with multiple nodules on body of 3 years duration. Initially, there was a painless nodule over the chest wall, followed in a few months by the development of more such papules and nodules on the trunk and limbs, which were ulcerating and discharging chalky white material on and off. There was no history of muscle weakness, arthralgia, photosensitivity, dysphagia, or Raynaud's phenomenon. Three other siblings were unaffected. The child was apparently given lots of milk up to 6 years of age, as informed by the parents.

**Figure 1:**
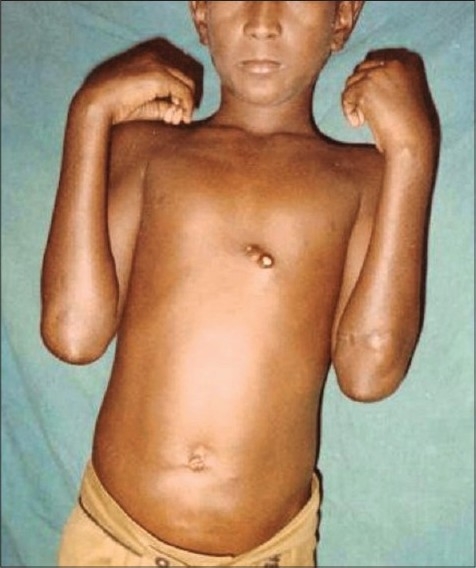
Papules 1 cm × 0.5 cm on the anterior chest wall, surmounted by chalky white material

**Figure 2:**
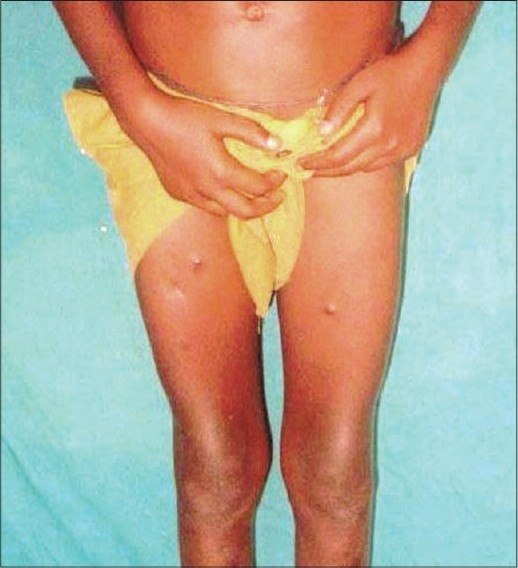
Papules and pitted scars over both thighs. Lesion on the right thigh discharging chalky material

Cutaneous examination revealed a large 3 cm × 1.5 cm, hard nodule on left forearm, three papules of 1 cm × 0.5 cm on the anterior chest wall, surmounted by chalky white material, along with a few scars in the vicinity [Figure 1], multiple non-tender, freely mobile papules and pitted scars over lower limbs, some discharging chalky material [Figure 2]. Other systems were clinically normal, except for angular stomatitis.

ESR was 50 mm/1^st^ hour, but all other hematological and biochemical parameters, including serum calcium and phosphorus levels, were normal.

X-Ray of chest revealed increased vascular markings, while X-Ray of left forearm showed a calcified subcutaneous nodule, not attached to bone.

**Figure 3:**
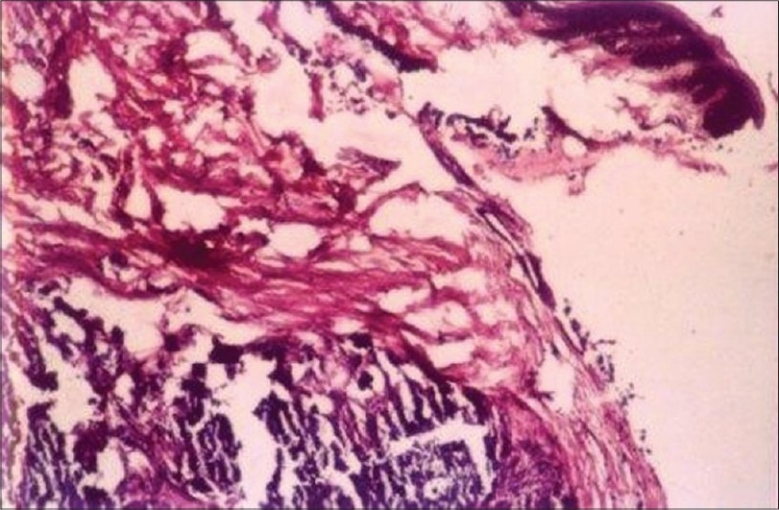
Skin biopsy showing calcification in subcutis (H and E, ×20)

HPE features were consistent with calcinosis cutis [Figure 3].

The term idiopathic calcinosis cutis is used when no obvious underlying cause can be identified for tissue calcification.

In present patient, dystrophic calcification, metastatic calcification, and iatrogenic calcinosis cutis were ruled out, respectively, by the lack of history of trauma, no preceding pathologic lesions at the sites of the papulo-nodular lesions, normal serum calcium and phosphorus levels, and absence of history of parenteral therapy. Idiopathic calcification can be wide-spread or localized and is described in the scrotum, penis, vulva and breast, with normal serum calcium levels. Tumoral calcinosis is a familial disorder associated with hyperphosphatemia.[1] Healthy adolescents can present with large calcified masses within subcutaneous tissue or muscles near large joints[2] without involvement of internal organs. Skin ulceration may occasionally be seen as in the present case.

The underlying mechanism of cutaneous calcification remains unknown. High levels of gamma carboxy glutamic acid (Gla), a unique amino acid, have been found in the calcified tissue and urine of patients with calcinosis.[3] Gla found normally in bones and teeth has calcium- and phospholipid-binding properties. Ectopic soft tissue calcification can be triggered if Gla is produced *de novo* at these sites.[4]

Diltiazem, colchicine, aluminium hydroxide, and warfarin are known to have beneficial effect in this condition.[5]

Indications for surgical treatment include painful masses, recurrent infection, ulcerations, functional impairment and cosmetic concerns.[6] However, in view of the multiplicity of lesions, surgery was not contemplated and our patient was advised frequent review to forestall any possible complications.
